# From disrupted routines to sustainable practice: integrating management sciences to navigate the innovation journey of precision imaging

**DOI:** 10.3389/fmed.2026.1965552

**Published:** 2026-10-20

**Authors:** Renaud Guignard, Bénédicte Geffroy, Valérie Fernandez

**Affiliations:** 1 Scintidoc, Montpellier, France; 2 Département Interdisciplinaire de Sciences Sociales (DI2S) d'IMT Atlantique, Laboratoire D'économie et de Management de Nantes Atlantique (LEMNA), Nantes, France; 3 Institut Interdisciplinaire de l’innovation (i3 CNRS), Telecom Paris, Institut Polytechnique de Paris, Palaiseau, France

**Keywords:** boundary work, dissemination & implementation, exnovation, innovation diffusion, precision imaging, professional jurisdictions

## Abstract

Precision medicine is reshaping medical imaging through artificial intelligence, quantitative biomarkers or hybrid modalities such as PET-CT. Yet these tools reach patients unevenly, and that unevenness is usually blamed on technical problems: interoperability, validation, trust. Drawing on management science and the sociology of professions, we propose a different explanation. What decides whether an innovation reaches patients equitably is not the technology, but the way it travels. We distinguish three ways of travelling. Diffusion is spontaneous spread through professional networks (letting it happen). Dissemination is spread deliberately directed towards a defined population (helping it happen). Implementation is the work of turning an adopted innovation into everyday routine (making it happen). Imaging innovations have travelled mainly by diffusion, which follows existing specialist density, funding rules and like-with-like professional networks; diffusion therefore reproduces the inequalities of access it inherits instead of correcting them. Three consequences follow. First, adoption hides three separate decisions taken by different people: acquiring the equipment, deciding what the examination actually contains, and deciding whether it is requested at all. Routine statistics count the first and the third; the second is never measured. Second, professional boundaries are held in place at once by regulation, by specialty jurisdiction and by everyday working relationships, so reforms acting on only one of them fail. Third, the medical report is where those boundaries meet: when it does not carry an integrated answer, the innovation stalls. A fourth point runs through all three: an innovation's value is not fixed before it travels; its construction is predicated on the expectations that precede it, and the practices through which it is utilised. Using a three-study programme on PET-CT in France as illustration, we argue that equitable and sustainable precision imaging is achieved not by buying equipment but by governing how innovations spread—including by actively retiring the low-value practices they were meant to replace, what management science calls exnovation.

## 1 Introduction: the innovation journey of precision imaging

Advanced diagnostic imaging has been credited with a substantial share of the recent decline in cancer mortality and with longevity gains achieved without commensurate growth in per-capita expenditure (1). Yet this aggregate value coexists with widespread variation in appropriateness—an estimated 20%–50% of imaging examinations are of low value (2)—foreshadowing the pivotal problem of this article: the translation of technological promise into clinical value is neither uniform nor automatic.

The evolution of precision imaging involves a restructuring process of what Abbott (3) defines as the system of professions, where different occupational groups compete for control over specific tasks. The introduction of AI-assisted imaging diagnostics, quantitative biomarkers or hybrid imaging (e.g., PET-CT) challenges the interpretation of complex data and requires a review of established routines in managing patient pathways. These tools promise to reshape diagnostics by augmenting the physician's clinical perspective with objective data.

Positron emission tomography combined with computed tomography (PET-CT) acquires two complementary images in a single session. The PET component maps metabolic activity: a radiopharmaceutical, most often a glucose analogue, concentrates in tissues with elevated turnover, so that tumours appear as focal areas of uptake. The CT component maps anatomy, locating those areas precisely and characterizing their size and their relations to adjacent structures. Each answers a different question—where is there abnormal activity, and what exactly is there—and the promise of the hybrid modality lies in answering both in one examination. Two features of the device matter for what follows. The CT component may be acquired either as a low-dose localizer, sufficient to position the metabolic findings, or as a full diagnostic study with intravenous contrast. France is unusual in that the two components fall within the competence of two distinct medical specialties: nuclear medicine (NM) and radiology. The device itself operates under a nuclear medicine authorization, while the qualifications required to perform acts using ionising radiation are set out in ASN decision n° 2020-DC-0694 of 8 October 2020 (homologated by the arrêté of 5 July 2021); the diagnostic scanographic act is not separately coded in the CCAM nomenclature when the CT is acquired as part of a hybrid examination. The practical consequence, which the argument below turns on, is not that the NM physician is forbidden to look at the CT, but that neither the authorization regime nor the payment nomenclature recognizes a diagnostic CT reading as part of the act being performed.

As Daly and Willis (4) argue, “*technological proliferation however, must occur in such a way that the skills of the medical profession remain esoteric, unavailable to other competing health workers”.* Authors posit that to reap the benefits offered by medicine, the medical profession must be preserved. Medical practice is never purely technical; it inherently relies on elements of “*indetermination”,* which include uncodifiable clinical judgment, personal experience, rules of thumb, and interpersonal skills.

Organizations with greater flexibility in financial and human resources, and a culture that embraces risk, are statistically more likely to adopt radical imaging innovations (5). The spread of new imaging technologies such as nuclear medicine (NM) imaging has historically been driven by the availability of specialists and reimbursement policies rather than clinical benefit, suggesting that technical prescriptions were firmly embedded in regional healthcare structures (6) and homophilous networks (7). This diffusion-dominant regime tends to reproduce the geography of advantage it inherits; once early adopters accumulate self-reinforcing “increasing returns” (8), that advantage becomes locked in. The deployment observed to be uneven is therefore not merely an accident of implementation, but the anticipated consequence of leaving spread to diffusion alone.

Throughout this article we treat “diffusion” and “dissemination” not as synonyms but as distinct modes of spread, following the distinction between “*letting it happen”,* “*helping it happen”* and “*making it happen”* (9):

- Diffusion denotes the largely passive, unplanned propagation of an innovation through informal professional networks—the epidemic logic of Coleman et al. (10) and Rogers (11).
- Dissemination denotes an active, directed effort to spread an innovation toward a defined population and to convert adoption into improved outcomes (12).
- Implementation, or embedding, denotes the organizational process through which an adopted innovation is introduced, integrated into local work processes, and progressively routinized (13, 14). This process may involve the appropriation of the innovation by local actors, who interpret, adapt, negotiate, and potentially transform it in relation to their work context (15–17).

In their seminal article, Greenhalgh et al. (9) distinguish diffusion as a largely passive process, dissemination as the active and deliberate effort to persuade and inform target audiences, and implementation as the organized work through which an innovation becomes embedded in practice. We propose to situate these three terms on a single analytical continuum; however, this is our own conceptual decision rather than a formulation explicitly advanced by Greenhalgh et al. It is grounded in the varying extent of deliberate labor required for an innovation's progression: none in diffusion, directed effort in dissemination, and sustained local work in implementation. This ordering provides the continuum with its analytical value here, insofar as the article's argument concerns the shift from processes that occur without purposeful intervention to those that depend on it.

One term used throughout this literature does not belong on the continuum. Adoption denotes a decision-event, the moment at which an actor or an organization commits to using an innovation, not a mode of travel. The three modes stand in different relations to it: diffusion produces adoption events incidentally, dissemination targets them deliberately, and implementation presupposes them. We therefore propose to decompose it. In a non-captant diagnostic service, adoption covers three decisions taken by different actors and located at different levels: (i) acquisition, where the organization commits to the technology (DiMaggio and Powell, 18); (ii) provision, where the imaging specialist determines what the examination actually contains (20); and (iii) referral, where it is incumbent upon the referring clinician to determine whether it is applicable (21, 22). In specialties whose members treat their own patients, the three decisions converge on the same clinician. Conversely, in medical imaging, equipment registries record acquisitions and activity statistics record referrals; provision leaves no trace in either. This is a statement about routine administrative data, not about research: provision can be and has been studied qualitatively, including in the empirical programme this article accompanies. The point is that the one decision which determines what the examination actually delivers is absent from the data on which diffusion is monitored and resources are allocated.

Implementation and appropriation are not synonymous, and the distinction matters for what follows. Implementation is assessed at the level of the organization—has the innovation become routine here? Appropriation is assessed at the level of the practice—what has the innovation become in these hands? We do not treat appropriation as a fourth mode: it is not a way of travelling but a way of relating to what has arrived, the question of “*what these objects, tools and devices become in the hands of the actors who put them to work*” (De Vaujany, 23; our translation). We retain it in one role only, as a qualifier of the depth of implementation, and we note that it falls outside a corpus assembled in English around diffusion in health systems, where the equivalent work travels under assimilation, embedding and normalization.

The innovation process is characterized by the development of new ideas or processes intended to yield partially stable outcomes, formulated by dynamic and loosely bounded groups of professionals within an evolving organizational context. Because innovation can encompass intangible artefacts, the notion of complex systems grounded in theory of hierarchy (24) is useful for a holistic, multi-level perspective on the innovation process, and narratives have proven a successful methodological approach for collecting qualitative data (25).

This theoretical contribution is the conceptual companion to a three-study empirical programme on the diffusion of PET-CT in France. The first study mapped a coherent nationwide expansion of PET-CT supply alongside persistent, unexplained regional disparities in its actual use (26). The second examined how nuclear medicine physicians appropriate a technology that straddles two professional jurisdictions, and found the decisive tension to lie within the specialty rather than between specialties (20). The third turned to the referring oncologists, radiation oncologists and surgeons who decide whether the examination is requested at all, and found adoption to be contested, relationally mediated and constrained by the report itself (22). Read together, these studies locate the obstacle to spread neither in the technology nor in any single professional group, but in the articulation between them.

Within the social science domain, the three-level framework has emerged as the prevailing approach to conceptualizing the location of change:

- The micro-level is the individual clinician and their immediate professional networks. In precision imaging, however, this level has two occupants rather than one. Imaging specialties are what Bergeron and Castel (21) call non-captant: they hold no patient population of their own and depend on referring clinicians to generate their activity. The split between provision and requesting therefore falls within this level, while acquisition sits at the next. Diffusion models that assume a single adopter cannot represent this split, and much of the unevenness of precision imaging originates at this micro-level fracture.
- The meso-level is the organization—the imaging department or hospital—with its teams, routines and resources.
- The macro-level is the wider health system—regulators, funding and reimbursement rules, and institutional norms. An imaging innovation must find its place at all three at once, which is why an analysis confined to any single level explains its uneven uptake only partially.

By reading the micro, meso and macro levels of imaging adoption as three points on the diffusion–dissemination–implementation continuum, this article advances a single theoretical proposition: a sustainable, human-centered and equitable precision imaging cannot be achieved by accelerating diffusion, because diffusion reproduces the inequities it inherits; it requires the deliberate, level-specific organization of dissemination and implementation — a managerial and jurisdictional accomplishment rather than a technical one.

## 2 Material and method

The contribution of this article is conceptual: it advances a framework for reading the innovation journey of precision imaging. The literature work described here is the method by which that framework was constructed—a transparent, bibliometric-informed synthesis of a deliberately cross-disciplinary body of work—rather than a systematic review reporting empirical findings.

### 2.1 Corpus construction

We assembled the corpus with Artirev® (Scanlitt SAS), an AI-assisted bibliometric application that automates the extraction, de-duplication, cleaning and conversion of citation data into valuable resulting maps that visually represent these data. The application integrates two complementary techniques, namely reference co-citation analysis (RCCA) and document bibliographic coupling analysis (DBCA; Walsh et al., 27), the purpose of which, in summary, is to answer a different question in plain terms:

- Co-citation analysis (RCCA) asks which earlier works later authors repeatedly cite together. Works consistently cited side by side form the field's shared intellectual foundations — its founding texts.
- Bibliographic coupling (DBCA) asks which recent papers draw on the same sources. Papers sharing many references form the field's current, active research streams.

For every node in the resulting maps—a cited reference in the RCCA, a source document in the DBCA—Artirev® computes a local hubness score, a centrality indicator that quantifies how strongly a work is connected to the other works within its own cluster or axis; the higher the score, the more that work structurally anchors its group (its “hub”).

Working from Scopus database queried through Artirev® on January 2026, we applied the Boolean query “diffusion” AND “innovation” AND “health” to the title and abstract fields. Following Rogers (11), this string targets the three constituents of diffusion—the innovation, its communication channels and the social (here, health) system—and screens out both generic clinical research unconnected to process innovation and management studies lacking a healthcare context.

We scoped the search to the research domains in which the diffusion of complex service innovations has been theorized—general management, organization studies, the sociology of professions and health-services research—rather than to a single discipline. We did not exclude health economics, whose analysis of adoption and access is integral to our argument (e.g., Miraldo et al., 7); we set aside only studies concerned solely with market forecasting or product marketing that carried no organizational, professional or health-system dimension.

The corpus is cross-disciplinary, and the outlets it spans differ in how their quality is conventionally assessed. We therefore did not build a ranking threshold into the query. We ordered our criteria rather than combining them:

- Peer review—condition of eligibility. No item outside peer-reviewed publication entered the corpus.
- Relevance to the diffusion of complex service innovations—condition of selection. Assessed by reading the abstract and, in doubtful cases, the full text. Every inclusion and exclusion decision was ultimately made on this criterion.
- Journal standing—complementary indicator, not a filter. Where an outlet is listed in the CABS Academic Journal Guide, its rating was consulted and a 2, 3 or 4 rating recorded as a positive signal; a low or absent rating was not sufficient ground for exclusion, and no item was retained on rating alone.
- Co-citation layer—outside the scope of these criteria. They govern the sixty source documents returned by the query, not the works those documents cite, which were never subject to retrieval criteria at all.

This procedure returned a working corpus of 60 documents (spanning 2001–2025 for the source articles), listed in full in Supplementary Material. Local hubness is therefore a relative, within-corpus measure of structural centrality rather than an absolute score or a global citation count: it ranks a work's importance inside this literature, so it can diverge from external citation counts—a work may anchor the corpus without being the most cited in absolute terms (as reported in the Results and Table 1).

**Table 1:** How the literature is structured, and how it maps onto the framework (Figure 1).

Bibliometric group *(number of works)*	What it represents	Most central works *(local hubness)*	Place in the framework
Founding texts Pole 1 (*n* = 10)	Adoption as the individual clinician's decision	Goes & Park 1997 *(1.38)*; Van de Ven 1999 *(1.22)*; Rogers 1983 *(1.14)*	Micro level
Founding texts Pole 2 (*n* = 7)	Adoption as a system-level, institutional process	Rogers 2003 *(1.36)*; Berwick 2003 *(1.13*); Greenhalgh 2004 *(1.03; 5,151 external citations)*	Macro level
Research stream 1 (*n* = 13)	Spreading & translating innovation into practice	Scarbrough & Kyratsis 2022 *(2.08)*; Keown 2014 *(1.65)*	Dissemination
Research stream 2 (*n* = 12)	Embedding innovation in the organization	Greenhalgh 2004 (*4.18*)	Meso/implementation
Research stream 3 (*n* = 9)	Professional networks & peer effects	Barrenho 2025 *(4.48)*; Barrenho 2021 *(1.36)*	Micro networks/diffusion

Corpus: 60 documents (Scopus, 2001–2025). Two complementary analyses were used: co-citation analysis (RCCA) identifies founding texts that later authors cite together; bibliographic coupling (DBCA) identifies current research streams that share sources. Local hubness measures how central a work is within its own group (higher = more central; values above ∼2 mark a dominant anchor, values near or below 1 a peripheral member). It reflects a work's structural position within this corpus, not its external fame—which is why a work can anchor a stream while having few outside citations [e.g., Barrenho et al. (38), hubness 4.48, is pivotal to its stream despite few citations].

Two features of this window deserve comment. It applies to the source documents returned by the query, not to the works they cite: because co-citation analysis operates on reference lists, the map reaches back well beyond its lower bound, and the most central works it identifies date from 1957, 1983 and 1988. The lower bound itself corresponds to the consolidation of complex service innovation as an object of health-services research in the early 2000s, immediately preceding the systematic review around which the field subsequently organized (Greenhalgh et al., 9).

Within this corpus, reference co-citation analysis (RCCA) identified the works most frequently co-cited across the retrieved literature—the field's shared theoretical foundation. The seventeen most central works resolved into two co-citation clusters: a micro-sociological paradigm (*n* = 10), anchored in Rogers (11), which frames adoption as an individual decision shaped by perceived relative advantage and compatibility with professional values; and a systems-and-dissemination paradigm (*n* = 7), anchored structurally in Rogers (28) and conceptually in Greenhalgh et al. (9) and Berwick (12)—the works that carry the diffusion-to-dissemination distinction itself—which foregrounds the institutional and system-level conditions that constrain or enable adoption. Sustainability and exnovation did not surface as a distinct coupling cluster: the field is organized around diffusion dynamics, service-organization embedding and active dissemination. We therefore treat that dimension not as an empirical axis but as the theoretical extension this article contributes at the macro level (29, 30).

### 2.2 From bibliometric map to conceptual framework

The relation between the bibliometric procedure and the framework proposed here requires statement, because the two are distinct operations and the second is not entailed by the first. Three steps may be separated:

- The first is bibliometric: co-citation and coupling identify relationships between publications and their structural positions within a corpus (31). This step produces groupings, not mechanisms.
- The second is interpretive: we read the publications in each grouping and characterized what they are about, an operation performed by the authors and not by the software.
- The third is theoretical: we constructed the diffusion–dissemination–implementation continuum, crossed it with the micro, meso and macro levels, and located the groupings within the resulting matrix.

Only the first step is bibliometric through the utilization of Artirev®. The second and third are qualitative and conceptual, and the correspondence between a coupling stream and a level of analysis is an interpretation we advance, not a result the method returns. Centrality within a citation network says nothing about processual relevance: a work may anchor the intellectual history of a field while saying little about how an innovation is negotiated in a department, and an ethnographic study of marginal citation weight may say a great deal. The clusters are therefore presented as heuristic structures that organized our reading, not as a representation of the intellectual organization of the field. The overall logic is abductive: we moved iteratively between the map, the concepts available in the literature, our reading of the source texts, and the emerging framework, revising each in light of the others (32, 33).

Three limitations follow. First, the working corpus contains sixty source documents, and the structures derived from it—two co-citation clusters and three coupling streams—are correspondingly small; we report no formal robustness checks on clustering thresholds, and a different resolution parameter would plausibly redistribute borderline members. Second, a citation-based approach privileges works that have accumulated citations, and therefore under-represents recent empirical studies and the qualitative and ethnographic literature that is central to our own argument; several works we mobilize in the discussion entered through reading rather than through the map. Third, the map cannot adjudicate between the theories it arranges. What the bibliometric step contributed, relative to an integrative qualitative review of the same corpus (34), was a defensible and reproducible basis for deciding which foundational works had to be engaged and for detecting that sustainability and exnovation form no coupling cluster of their own—an absence that motivated the theoretical extension we propose at the macro level, and that a purely qualitative reading would have been unlikely to establish.

## 3 Results

Together the RCCA and DBCA analyses yield two founding poles and three research streams: (i) the poles identify the levels at which precision imaging innovation must be understood—from the individual clinician to the whole health system; (ii) the streams identify the movement an innovation makes over time—from initial spread to durable, routine use. Crossing levels with movement produces the framework proposed here (Figure 1): a diffusion-dissemination-implementation continuum read across the micro, meso and macro levels. Table 1 summarizes each group, its anchor works and its place in the framework.

**Figure 1:**
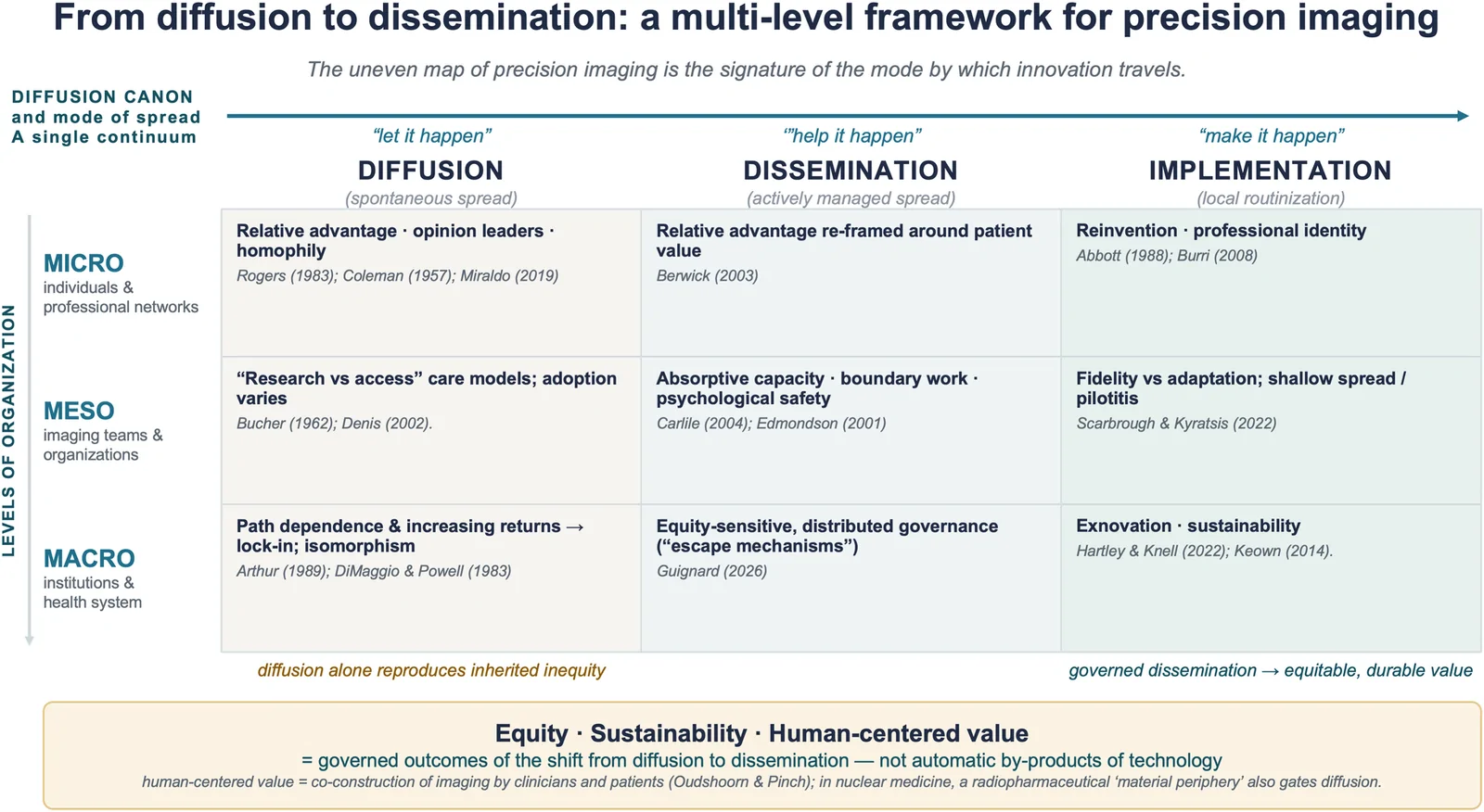
conceptual matrix summarizing the article's framework. Three columns represent modes of innovation spread—diffusion (spontaneous), dissemination (actively managed) and implementation (local routinization)—and three rows represent levels of analysis: micro (individual clinicians and professional networks), meso (imaging teams and organizations) and macro (institutions and health-system governance). Each cell names the management-science mechanism operating at that intersection, from relative advantage and opinion leaders at the micro level, to absorptive capacity and boundary work at the meso level and isomorphism, path dependence and exnovation at the macro level. A fourth proposition, stated in the Results, cuts across the matrix rather than occupying a cell: each mode is also an operation of valuation, whose residue—expectations, value propositions, inscribed scripts—forms the environment in which the next is performed. The upper horizontal arrow marks the shift from letting spread happen to deliberately governing it for equitable, sustainable outcomes. The left vertical arrow renders the second proposition stated in the Results: a single professional demarcation may be upheld simultaneously by three superposed strata—relational at the micro level, jurisdictional at the meso level and regulatory at the macro level.

### 3.1 The two foundations: the individual adopter vs. the system

Co-citation analysis (RCCA) showed that the field rests on two sets of founding texts—best read not as rival schools but as the two ends of a single question: at what level does adoption actually happen?

The first foundation (*n* = 10 works) treats adoption as an individual decision. Anchored in the classic diffusion literature—Rogers (11), Coleman et al. (10) and Van de Ven et al. (43)—it explains uptake through how each clinician perceives an innovation: its relative advantage, and its compatibility with their values and professional identity.

The second foundation (*n* = 7 works) treats adoption as a system-level process. It is anchored in the two works that introduce the distinction at the heart of this article [Greenhalgh et al. (9) and Berwick (12)] with Escarce (35) and Rogers (28), the latter carrying the highest local hubness of the pole (Table 1). It foregrounds the institutional conditions that enable or block adoption, and the shift from spontaneous spread to actively managed dissemination. The two editions of Rogers (11, 28) anchor opposite poles; this reflects differing citation habits between the micro-sociological and the systems literatures rather than a theoretical divergence between the editions.

The field's enduring weakness is that these two foundations are usually studied apart: work on individual choice rarely meets work on system-level constraint. A framework able to explain why precision imaging spreads unevenly must hold both together—the purpose of the micro-to-macro axis in Figure 1.

### 3.2 The three research streams: from first spread to routine use

Bibliographic coupling analysis (DBCA) sorted the corpus into three active research streams that together trace an innovation's path from first appearance to embedded clinical routine.

- Stream 1- spreading and translating innovation (*n* = 13 works):

Anchored by Scarbrough and Kyratsis (14) and the “science of delivery” literature (29, 36), this stream treats innovation not as a one-off installation but as continuous work to translate a technology's technical capability into everyday clinical utility—the work of actively disseminating AI tools or new tracers, not merely acquiring them.

- Stream 2—embedding innovation in the organization (*n* = 12 works):

Anchored by Greenhalgh et al. (9), this stream shifts attention from the individual to the department, showing that whether an acquired technology is actually used depends on the organization's capacity to absorb it and to renegotiate professional roles around it.

- Stream 3—professional networks and peer effects (*n* = 9 works).

Anchored by Barrenho et al. (37, 38) and Rochefort (39), this stream examines how diffusion actually travels between clinicians—through peer influence and professional networks—and provides the empirical grounding for the micro-level analysis that follows.

### 3.3 The framework: a diffusion–dissemination–implementation continuum across levels

The framework yields the article's central proposition. Left unmanaged, diffusion is not merely uneven but self-reinforcingly inequitable: because it travels along homophilous professional networks (7) and pre-existing infrastructures of specialist density and reimbursement (Bouckaert and Leroy, 6—in the period they study), and because early advantages accumulate as increasing returns (8), it reproduces the geography of advantage it inherits. Equity, sustainability and human-centered value are therefore not automatic by-products of technological progress but outcomes of a deliberately governed shift from diffusion toward dissemination and implementation—organized differently at each level. This reframes, at the conceptual level, the very gap our companion empirical study could document but not resolve (26), and it structures the discussion that follows, which specifies what that shift requires at the micro, meso and macro levels in turn.

A second proposition follows from the framework and qualifies the first. Boundary work is not confined to the meso level. The same demarcation may be simultaneously regulatory (authorization regimes, act-coding rules), jurisdictional (the demarcation of legitimate practices within and between specialties) and relational (trust, reciprocity and availability in the referral dyad)—one stratum at each level of organization, macro, meso and micro respectively. We propose that the resistance of a professional boundary to innovation increases with the number of strata that sustain it: where a single demarcation is upheld at all three levels at once, no single-level intervention can displace it. This is why the shift from diffusion to deliberate dissemination must be organized at the three levels jointly rather than sequentially, and it explains why purely regulatory reforms and purely relational devices have both proved disappointing in hybrid imaging.

This proposition carries a scope condition worth stating. It concerns innovations whose value depends on competences held by more than one professional jurisdiction. An innovation internal to a single jurisdiction encounters no superposed boundary, and may spread through diffusion alone. The condition is related to, but not identical with, the distinction between sustaining and disruptive innovation (40), which is defined by performance trajectories and market entry rather than by jurisdictional crossing: a disruptive innovation may leave professional boundaries intact, and a sustaining one may cross them. What determines whether the three strata bind is not the magnitude of the technical change but whether the innovation requires two professional groups to act on one another's terrain.

A third proposition concerns the depth of implementation rather than the distance travelled. Completing the three-term continuum proposed here guarantees that an innovation has become routine; it does not guarantee that it has become what it was designed to be. We therefore propose that the outcome of an innovation journey be assessed on two axes rather than one: the distance travelled along the diffusion–dissemination–implementation continuum, and the mode of appropriation reached at the point of arrival—appropriation being retained here, as stated above, as a qualifier of depth rather than as a fourth mode of spread. Innovations that complete the continuum under a neutral mode of appropriation are, on aggregate indicators, indistinguishable from successes—which is the deeper reason why volume-based measures of diffusion mislead.

A fourth proposition concerns what the continuum does to the value of the innovation that travels it. The first three have been stated as though an innovation of given value moved through a social world that receives it. The sociology of translation holds that the two are produced together: an innovation becomes established as its promoters problematize a situation and enrol the actors whose alignment it requires (41), while the object itself inscribes a script for who does what (42). We therefore propose that each mode of spread is also an operation of valuation, whose criteria become the inherited condition of the next. Diffusion still reproduces the inequities it inherits; what it inherits is in part shaped by what its earlier passage has deposited.

Taken together, these four propositions constitute the organizational lens this article proposes: a reading of imaging innovation in which the mode of spread and the level of organization are considered jointly rather than in isolation. Section 4 applies it in turn to the micro level, where the adoption decision is split; the meso level, where boundaries are negotiated and the report is designed; and the macro level, where diffusion is governed and legacy practice retired.

## 4 Discussion: navigating the innovation journey in precision imaging

As suggested by the “*innovation journey*” framework (43), the diffusion of precision medicine innovations is a non-linear, complex process. It is contingent upon the interplay between individual professional acceptance, organizational learning routines, and macro-level institutional pressures.

### 4.1 Micro-level: redefining relative advantage through social networks

As noted in the introduction section, adoption is fundamentally socio-technical and relies on the individual perception of innovation attributes (11). The literature reveals a critical tension here: clinicians often resist innovation because they perceive a lack of compatibility with their professional jurisdictions and existing values (44). Read through the sociology of translation, what the referring clinician evaluates is not a fixed relative advantage but the outcome of an interessement that may or may not have been performed.

#### 4.1.1 From technical efficiency to human-centered value

Human-centered value is not a supplement to technical performance but a condition of adoption. Person-centered strategies take hold only where clinicians recognize the innovation as compatible with their sense of their own role, and not merely with their workflow (44).

The utilization of a diffusion-and-dissemination account has the potential to remain clinician-centric, tracing how innovations travel between professionals while leaving patients as passive endpoints. Yet imaging value is not fixed at the scanner; it is co-constructed in the clinical encounter and along the care pathway, as referring physicians, patients and imaging specialists jointly determine what a scan means and what follows from it.

Reading the human-centered imperative through the co-construction of users and technologies (45) makes patients and referrers active participants in the innovation journey rather than its recipients; it also warns that technologies are implicitly designed for those with the most resources, access and voice, so that under-served groups risk being tacitly constructed as “non-users”. Giving concrete content to shared decision-making and patient-reported outcomes in imaging is, in this sense, inseparable from the equity agenda.

#### 4.1.2 The pivot of social networks

Because uncertainty is high in precision medicine, adoption is fundamentally social. Physicians are socially embedded actors, and the decisions they take about a technology are shaped by their position in a professional field as much as by the technology's properties: information about an innovation travels through professional networks (10), while imaging apparatus itself functions as a resource in the competition for standing among specialists (46). Early adoption is often driven not by proven technical benefits, but by a strategic search for social gains and legitimacy, where peripheral actors seek to improve their status by becoming “*exemplary users*”. This suggests that in high-tech fields like robotic surgery, diffusion may be initially propelled by “*discursive persuasion*” and reputation-building rather than purely rational economic assessments (47).

Contrary to the assumption that institutional dictates drive change, evidence suggests that individual clinicians and their professional networks are powerful agents of diffusion. In the context of a surgical innovation, hospital-level factors accounted for less than 1% of the variation in adoption, while surgeon-level characteristics explained close to half of the diffusion of this technology (variance decomposition of share of total elective colorectal cancer colectomies performed laparoscopically: 46.6%). The size of a surgeon's network (connectivity) is positively correlated with the likelihood of adoption. Adoption is also heavily influenced by peer effects (contagion), where a surgeon's likelihood of adopting a new technique increases significantly if their peers and network connections have already done so. Social networks are independent and powerful drivers of diffusion, facilitating the spread of innovation through information exchange and social contagion (37).

This evidence, however, comes from a captant specialty: the surgeon adopts a technique and performs it on their own patients, within their own institution, and without prior authorization of the equipment. Diagnostic imaging differs on all three counts. The near-absence of hospital-level variance observed in colorectal surgery therefore cannot be transposed without qualification: in imaging, regulatory, institutional and network determinants operate jointly rather than as alternatives, and this joint operation is one reason why the diffusion regime is so difficult to redirect.

Opinion leaders are the usual explanatory device usually invoked at this point, and the evidence about them is more equivocal than the enthusiasm for it suggests. Reviewing two national programmes for the transfer of evidence into practice, Locock et al. (48) report that systematic support for their use as an intervention is limited, that effects are mixed, and that what such individuals actually do resists replicable description, since who counts as one differs from setting to setting. Two of their findings bear directly on the framework proposed here. First, expert and peer opinion leaders carry weight at different moments: authority over the evidence matters while the question is whether the innovation is worth having, whereas influence over colleagues matters once the question becomes how to work it into a routine—a distinction that maps onto the passage from dissemination to implementation, and one whose two halves may be in dissonance. Second, and more often neglected, opinion leaders may be ambivalent or hostile: a credible local sceptic is as consequential as an advocate, and formal designation as a champion may itself erode the influence it was meant to mobilise. These networks serve as one mechanism for reducing the innovation risk perceived by individual adopters (49). Conversely, professional networks are often characterized by homophily, a tendency for individuals to engage with peers of analogous seniority and specialization. This phenomenon is often characterized by the formation of information silos, where knowledge is disseminated within closed circles of professionals and fails to diffuse to the wider medical community, reinforcing the gap between innovator hubs and peripheral institutions (7).

#### 4.1.3 The gatekeeper and the interdependence of adoption decisions

Peer-effect models assume that the adopter and the operator of an innovation are the same person: the surgeon who adopts laparoscopy performs it on their own patients. Diagnostic imaging breaks this assumption. Two of the three decisions distinguished above—provision and referral—are held by different professionals in different jurisdictions, and they are coupled by a single artefact: the report. Where that artefact fails to carry an integrated answer, the coupling degrades. Referring clinicians then read the NM specialist's restraint on CT interpretation as a competence deficit, whereas the same NM specialist reads this restraint as legitimate differentiation—an asymmetry of framing that leaves neither party with an endogenous reason to change (22). Social exchange theory clarifies the consequence (50, 51); referral rests on an unspecified obligation of reciprocity, and trust is reported to attach to the individual reporter rather than to the modality or the specialty. At this level, relative advantage is therefore not a property of the technology but the outcome of a cross-jurisdictional exchange, which is why interventions aimed at the imaging specialist alone, or at the referrer alone, tend to under-perform.

### 4.2 Meso-level: disrupted routines, professional identity and organizational embedding

#### 4.2.1 Disrupted routines and professional identity

Innovation is not merely a tool; it is an occasion for professional and routine change. The introduction of complex imaging, such as the first CT scanners, forced a renegotiation of expertise between radiologists and technicians, altering the distribution of expert knowledge and threatening established dominance hierarchies (52). More advanced technologies such as quantitative MRI (qMRI) or hybrid operating rooms also necessitate “*boundary work*” (53) — the negotiation and reconstruction of professional roles between radiologists, surgeons and technicians (54). This process is recursive: emerging practices drive changes in boundaries, while new boundaries stabilize practices (55).

Boundary work is also, and sometimes primarily, internal. Bucher and Strauss (56) describe professions as coalitions of segments rather than unitary actors. In French nuclear medicine, the appropriation of hybrid imaging has not produced a frontal contest with radiology—radiologists, ten times more numerous and facing rising demand, do not experience the encroachment as a threat—but a division within the medical imaging specialty itself, between a closure strategy that confines expertise to the metabolic domain and an opening strategy that incorporates radiological interpretation in order to strengthen the diagnostic demonstration before referrers (20). Absorptive capacity at the meso level is thus conditioned by an unresolved intra-professional settlement, and not only by inter-professional negotiation.

#### 4.2.2 The hard core, the soft periphery and a material periphery

Management literature suggests that technology implementation often fails not because of the technology but because it disrupts routines. The fusion of anatomical radiology and functional nuclear medicine required by hybrid imaging blurs traditional roles and creates friction (20). Complex innovations possess “fuzzy boundaries”—a “hard core” (the technology) and a “soft periphery” (the organizational structures required for implementation) whose adaptiveness is decisive (9).

This model, however, requires an amendment in nuclear medicine. Beyond the hard core and the organizational soft periphery, radiopharmaceutical-based imaging depends on a material and logistical periphery that has no equivalent in most software or device innovations: tracers with short half-lives, cyclotron and radiochemistry capacity, radioprotection regimes, and supply chains whose fragility can halt diffusion irrespective of clinical demand or organizational readiness. As nuclear oncology moves toward theranostics, this material periphery—not only professional acceptance—will increasingly gate the equitable dissemination of precision imaging (26). This constraint is not peculiar to radiopharmaceuticals. Sequencing capacity and reagent supply in molecular diagnostics, and computational infrastructure in AI-assisted imaging, impose material peripheries of the same kind: logistical dependencies that can halt diffusion irrespective of clinical demand or organizational readiness.

#### 4.2.3 The report as the boundary object of hybrid imaging

The management literature usually locates boundary objects in pathways, protocols or method cards (55). Within the domain of medical imaging, the conventional boundary object is of a more pragmatic and consequential nature: the report. It is simultaneously the product of a regulatory constraint—under the French act nomenclature, the CT component of a PET-CT examination is not separately coded or reimbursed as a diagnostic radiological act, and the device operates under a nuclear medicine authorization rather than a radiological one—the expression of a jurisdictional strategy, and the medium of the referral relationship.

This is also where the innovation's script is inscribed. In Akrich's (42) terms, a technical object configures its users: it establishes who is competent to say what, and what the recipient is expected to infer. A report that separates metabolic from anatomical findings does not merely fail to transfer knowledge across the boundary; it inscribes a distribution of competences and configures the referring clinician as someone who will require a further examination. Designing the report is an act of inscription: it scripts its users and the competences attributed to them.

In Carlile's terms (54), the fragmented PET-CT report operates as a syntactic repository, at best as a semantic standard, but not as a pragmatic object able to transform knowledge across the boundary (57). What managers diagnose as a formatting deficiency may therefore be an active mechanism of boundary maintenance rather than a design imperfection. Structured, image-rich reporting (SIRR) is, on this reading, not a documentation improvement but the deliberate design of the artefact on which cross-jurisdictional coupling depends—which also implies that imposing a template without addressing the underlying asymmetry of framing would yield formal compliance without changed practice.

If exnovation depends on the quality of the boundary object, that quality has to be specified. Three properties are inherited from the literature:

- Plasticity: the artefact accommodates the local interpretive needs of each group, so that the referring clinician and the imaging specialist each find in it what their own practice requires (Star and Griesemer, 57).
- Robustness: it maintains a common referent across those groups, so that they are demonstrably discussing the same patient and the same finding.
- Transformative capacity: it obliges each party to alter its own knowledge rather than merely receive the other's, which is what distinguishes a pragmatic boundary object from a repository (Carlile, 54).

We propose a fourth property, which we have not found articulated in this literature and which the exnovation argument requires. We call it decidability: the artefact must contain enough to close the clinical question that prompted it, so that no residual uncertainty justifies ordering the examination it was meant to replace. The first three properties concern what an object allows different groups to do with it; decidability concerns what it allows them to stop doing. For PET-CT this is concrete. A report is decidable when it integrates metabolic and anatomical findings in a single synthesis rather than juxtaposing them, reports the measurements the referring clinician needs for staging or response assessment, answers the question actually asked, and states explicitly what does not require further imaging. An object may be plastic, robust and transformative and still fail on this last property—which is precisely the configuration we observe, and precisely why the legacy examination survives.

#### 4.2.4 The implementation gap

The introduction of an innovation expands the options available to clinicians. In the absence of a shared understanding of the appropriate use, that latitude produces “unwarranted variation”—differences driven not by patient needs but by physician-specific factors such as practice style, risk aversion and school of thought (7). The same deficit has a second and apparently opposite expression: where a department has made an innovation work but cannot articulate what makes it work, its success does not travel, and local innovators succeed within their own setting while failing to disseminate beyond it—“pilotitis” (Scarbrough and Kyratsis, 14).

Unwarranted variation and pilotitis are not contrary problems but one problem seen from two angles, and together they compose the implementation gap. What fails to travel, in both cases, is not the technology but the knowledge of why it works—which is precisely what diffusion, left to itself, does not carry. The inertia is compounded when innovations survive as temporary projects serving a managerial rather than a clinical function, allocating resources or generating enthusiasm without altering core workflows (58): there, no transmissible account is produced at all.

#### 4.2.5 Absorptive capacity, psychological safety and team learning

Successful assimilation requires absorptive capacity—the organizational ability to identify and assimilate new knowledge (9)—which is not passive but requires active team learning, and a climate of “psychological safety” in which teams can experiment with new workflows without fear of judgment or damage to professional identity (Edmondson et al., 62). In medical imaging, introducing innovations such as quantitative biomarkers must be accompanied by “learning collaboratives” (36) that allow imaging specialists, technicians and physicists to renegotiate their roles; without such investment in the soft periphery, the hard core of the technology remains underutilized. This has been observed for PET-CT in France (20): fusing anatomical (CT) and functional (PET) data demands more than juxtaposed workstations—it requires the negotiation of knowledge boundaries (54).

#### 4.2.6 Fidelity vs. adaptation

A central management challenge is the tension between fidelity to an evidence-based protocol and the need for local adaptation (12, 39). Top-down compliance mandates frequently yield a “shallow spread” in which innovation is not disseminated extensively (14). Successful implementation requires “reinvention”, whereby local teams adapt the innovation to their infrastructure (11); yet a lack of “principles-knowledge”—understanding why an innovation works—can strip a strategy of its effectiveness and cause drift (59). Sustainable models therefore rely on boundary objects such as adaptable clinical pathways—for example the method cards used in hybrid ORs—that standardize core components while allowing local flexibility (55).

### 4.3 Macro-level: from isomorphism to equity-sensitive, sustainable dissemination

The uneven deployment of medical imaging innovations is also shaped by the macro-environment. Hospitals frequently adopt technologies through institutional isomorphism—the pressure to emulate leading “prestige” institutions to gain legitimacy (mimetic isomorphism) or to comply with regulatory mandates (coercive isomorphism)—rather than to meet specific clinical needs (18, 49).

This creates a risk of “cosmetic adoption”, where expensive equipment is purchased to signal modernity but is not integrated into care. The value propositions on which acquisition decisions draw offer little resistance to that risk. Manufacturers' framings combine clinical, technical, structural and human claims, and mobilize values—prevention, efficiency, security, ease of use—that resonate across the whole range of health system stakeholders; what they leave out is how the innovation will alter the delivery of care, and they obfuscate the trade-offs it entails from a system perspective, presenting goals that may conflict, such as earlier detection and cost reduction, as compatible (19). Such framings bolster expectations while being difficult to counter, given how little evidence is available at the point of decision. The omission is precisely the one this article identifies: the value proposition addresses acquisition and is silent on provision.

Hybrid imaging extends the notion of cosmetic adoption from the acquisition to the act itself. Activity statistics count PET-CT examinations without recording whether the CT component was acquired diagnostically and interpreted, so an examination in which the CT serves only for attenuation correction and anatomical localization is registered identically to a fully integrated one. Measuring diffusion by volume therefore conceals a second gap—between the hybrid act and the hybrid act in name only—and no equity or exnovation agenda can be operationalized without an indicator of effective integration alongside the count—that is, an indicator of provision. Cosmetic use is the macro-level statistical signature of a neutral mode of appropriation at the meso level (17): the two describe the same phenomenon from the two ends of the system.

France's PET-CT trajectory illustrates the mechanism. Early, context-driven advantages in certain regions became entrenched as adoption and multidisciplinary-team discussion fed back into further adoption—Arthur's (8) “*increasing returns*”—widening the gap with regions of slower initial uptake (26). Diffusion, left to itself, is therefore not merely uneven but self-reinforcingly inequitable, which is precisely why an equity-sensitive dissemination has to be deliberately governed rather than awaited. The equity argument gains a further edge here. If expectations orient investments and institutional arrangements before evidence is available (67), regions that adopted early did not merely accumulate equipment; they accumulated the criteria by which that equipment's value is subsequently judged. The map of regional disparity is, in part, a map of where the innovation was successfully problematized first.

Burri's (46) ethnography of imaging departments in Europe and the United States gives this mechanism a concrete form. Scanners function as status objects, compared by magnet strength as much as by cost; one department head recalls securing the funding before informing his hospital, and recalls that in the acquisition of a new scanner “power, money, and status” were the underlying objects of all the discussions—whereupon colleagues in neighbouring departments announced that they too would buy one. Mimetic isomorphism is thus observable at the scale of the individual purchase. Burri further notes that the symbolic status of the apparatus persists after installation, since the department that houses it governs who may use it and when—a configuration that anticipates the French case, where the hybrid camera sits within nuclear medicine.

Sustainability adds a further imperative. The current paradigm often conflates innovation with addition; a genuinely sustainable model requires governance that rewards exnovation (60) and defined as the active decommissioning of obsolete or low-value legacy practices (Kimberly, 61), rather than layering new technology on old. Studying the diffusion of innovation across eight health systems, Keown et al (29), describe the result of not doing so as a “paleontological stratification of practices”: layering innovations on top of multiple surviving practices, increases costs, reduces productivity, and undermines quality and outcomes. They also point to an instrument for the opposite course, the “do not do” list of the National Institute for Health and Care Excellence (NICE), a set of guidelines which formalizes the evidence base for abandoning superseded procedures and thereby supplies a rationale against their continued use. What makes such instruments hard to apply is documented by Hartley and Knell (30): organizations more often keep the superseded innovation on the books and quietly ignore it, and termination is resisted both by innovations “doomed to success” that no one will be seen to abandon and by stakeholders who gain from the status quo.

Framing sustainability through exnovation also reconnects the argument to the environmental agenda of precision imaging: low-value and redundant examinations carry a material footprint—energy, contrast media, radiopharmaceutical production and waste, equipment life-cycles—so decommissioning obsolete practices is not only an economic and organizational act but an environmental one, operationalizing the appropriateness criteria, low-dose protocols and equipment life-cycle optimization that sustainable radiology requires.

The PET-CT case supplies a direct illustration of exnovation failure. In specific contexts, such as in France, referring clinicians frequently request both the hybrid examination and a conventional CT, and imaging specialists describe them as strongly reluctant to abandon the latter; the fragmented report itself generates additional examinations to answer the questions the PET raised (22). The new modality is layered onto the legacy one rather than replacing it. This suggests that exnovation is not solely a macro-level governance act: it fails at the macro level partly because the meso level artefact gives referrers no grounds on which to abandon the legacy test. Decommissioning low-value practice therefore depends on the prior quality of the boundary object that is supposed to render it redundant—a cross-level dependency that governance instruments acting on incentives alone cannot resolve.

## 5 Future research: unpacking the black box of implementation through qualitative management research

To bridge the gap between the technological promise of precision medicine and its uneven clinical reality, future research must expand beyond the “variance research” that has historically dominated the diffusion field (11). Previous studies have largely relied on aggregate provider-level data, which fails to disentangle the complex supply-side factors (physician agency, peer effects…) from demand-side patient characteristics (7).

While quantitative metrics effectively track adoption rates and health outcomes, they often treat the implementation process as a black box, failing to explain how and why innovations succeed or fail in specific organizational contexts (9). To extend innovation in precision medicine effectively, we propose a paradigm shift toward the extensive utilization of qualitative studies grounded in management science.

### 5.1 Illuminating micro-dynamics and team routines

Quantitative data might correlate hospital size with adoption, but only qualitative inquiry can reveal the collective learning processes required to make these technologies work. As demonstrated by Edmondson et al. (62) in cardiac surgery, qualitative field research is essential to identify how psychological safety and leader inclusiveness allow teams to disrupt routines safely. Future research in precision imaging must employ ethnographic and observational methods to understand how interdisciplinary teams negotiate the boundary work required to operate complex technologies, and, given the split adoption decision described above, must extend observation to the referral dyad rather than confining it to the imaging department.

### 5.2 Analysis of legitimation and value narratives

The adoption of precision imaging technologies is heavily influenced by the legitimation narratives constructed by professionals to navigate the uncertainties of new technologies. While technical efficacy is a prerequisite, it is the professionals who must socially construct the utility of these tools to establish their jurisdiction. Mignot (63) illustrates this through the sociogenesis of interventional radiology, identifying how practitioners deployed specific legitimation strategies, ranging from the creation of a distinct semantic label to the establishment of learned societies, to transform their professional identity from passive diagnosticians to active therapists. Similarly, interview-based studies in reproductive medicine have shown how professionals use narrative strategies to legitimize add-on technologies even in the absence of robust randomized controlled trials (64). Applying these methodological lenses to precision imaging would allow us to understand how soft evidence and opinion leaders influence the acceptance of innovation where hard evidence may be evolving or contested (48).

### 5.3 Anticipating future roles and responsibilities

Finally, qualitative methods such as co-creation workshops and scenario drafting are vital for anticipatory governance in precision medicine. Rather than reacting to technological disruption, these methods allow stakeholders to proactively imagine post-digital futures. For example, Van Hees et al. (65) utilized Responsible Research and Innovation (RRI) qualitative designs to help MRI professionals co-create future scenarios regarding quantitative imaging. This approach revealed how forward-looking responsibilities and professional identities must shift to accommodate new workflows. Furthermore, in-depth case studies can reveal how innovation projects survive not because of their technical success, but because they serve latent managerial functions, such as generating enthusiasm or correcting system failures, that quantitative metrics would classify as inefficiencies (58).

### 5.4 Managerial implications and actionable paradigms for precision medicine

To bridge the gap between the promise of precision medicine and the reality of clinical practice, we propose three actionable managerial paradigms grounded in management science:

- Design for compatibility: healthcare leaders must frame innovations not just as technical upgrades, but as tools that enhance the professional identity and relational capacity of the professional (micro-level).
- Invest in implementation teams: resources must be allocated not only to hardware but to the implementation infrastructure—creating safe spaces for interdisciplinary teams to relearn routines and adapt protocols to local contexts (meso-level), and to the co-design, with referrers, of the boundary object through which the innovation is delivered.
- Govern for value: policy incentives must shift from rewarding the acquisition of novel technology (isomorphism) to rewarding its sustained, appropriate use and the de-implementation of low-value legacy practices (macro-level), which presupposes indicators of effective integration rather than of volume.

By integrating these management science perspectives, medical imaging can navigate the complex “*innovation journey*” (43), ensuring that technological advancement translates into sustainable, human-centered patient care.

## 6 Conclusion

Precision medicine advances by delegating ever finer discriminations to specialized diagnostic services, and imaging is where that delegation is most visible. Its uneven clinical translation is best understood not as an unfinished diffusion but as a diffusion that has done exactly what diffusion does: followed the lines of least resistance. Reframing the problem as one of mode—diffusion vs. dissemination vs. implementation—allows management science to specify what a corrective would entail at each level, and to state plainly that equity and sustainability are governance choices, not by-products of technological progress.

The practical consequence is measurable, and uncomfortable. A health system can count the (PET)scanners it has installed and the examinations it has performed and conclude that an innovation has arrived. Neither figure records what those examinations actually contained. An innovation can therefore complete its journey—acquired, requested, routine—and still deliver a fraction of what it promised, with nothing in the statistics to show it. Closing the implementation gap begins by measuring the decision that no one currently measures. What that decision involves—boundary work, legitimation strategies, learning routines—is not visible in aggregate data; it requires qualitative inquiry into how professionals negotiate the technologies they are made to share. What the statistics fail to record is therefore not only a decision but the moment at which the innovation's value is actually made.

The scope of this claim extends beyond imaging. The mechanisms specified here operate wherever three conditions hold: an innovation whose value depends on combining competences held by distinct professional jurisdictions; a delivering service that holds no patient population of its own and therefore depends on referral; and an artefact—a report, a panel result, an algorithmic output—through which the finding must cross a professional boundary before it can inform a decision. Molecular diagnostics and genomic tumor boards, pathology in precision oncology (66), and AI-assisted decision support all share that structure, and should be expected to display the same signature: rapid apparent spread, persistent territorial inequity, and a promise of integration that the delivering artefact does not carry.

Nuclear oncology offers the most immediate opportunity to test this. Radioligand therapy displaces the most fundamental demarcation of all—that between diagnosis and treatment—and converts the imaging specialist from a diagnostic consultant into a therapeutic partner managing toxicity and long-term follow-up. If the account proposed here is correct, and if the unevenness of PET-CT stems from superposed boundaries rather than from the technology, this transition should encounter resistance of at least comparable intensity, and its dissemination should depend on whether the relational, jurisdictional and regulatory strata are addressed together. Observing it prospectively, rather than reconstructing it afterwards, is the most direct test this framework admits. Precision medicine will not be limited by what its technologies can detect. It will be limited by what its professions are able to share.

## Data Availability

The original contributions presented in the study are included in the article/Supplementary Material, further inquiries can be directed to the corresponding author.
